# Molecular Mechanisms of Chondrocyte Proliferation and Differentiation

**DOI:** 10.3389/fcell.2021.664168

**Published:** 2021-05-28

**Authors:** Hui Chen, Xiao-Ning Tan, Shi Hu, Ren-Qin Liu, Li-Hong Peng, Yong-Min Li, Ping Wu

**Affiliations:** ^1^Hunan University of Chinese Medicine & Hunan Academy of Chinese Medicine, Changsha, China; ^2^The Affiliated Hospital of Hunan Academy of Chinese Medicine, Changsha, China; ^3^Department of Pharmaceutical Engineering, School of Chemical Engineering, Xiangtan University, Xiangtan, China; ^4^Center for Bionic Sensing and Intelligence, Institute of Bio-medical and Health Engineering, Shenzhen Institutes of Advanced Technology, Chinese Academy of Sciences, Shenzhen, China; ^5^School of Computer, Hunan University of Technology, Zhuzhou, China

**Keywords:** chondrocyte, transcription factor, growth factor, Wnt, TGF-β, FGF, Ihh Notch

## Abstract

Cartilage is a kind of connective tissue that buffers pressure and is essential to protect joint movement. It is difficult to self-recover once cartilage is damaged due to the lack of blood vessels, lymph, and nerve tissues. Repair of cartilage injury is mainly achieved by stimulating chondrocyte proliferation and extracellular matrix (ECM) synthesis. Cartilage homeostasis involves the regulation of multiple growth factors and the transduction of cellular signals. It is a very complicated process that has not been elucidated in detail. In this review, we summarized a variety of signaling molecules related to chondrocytes function. Especially, we described the correlation between chondrocyte-specific regulatory factors and cell signaling molecules. It has potential significance for guiding the treatment of cartilage injury.

## Introduction

Articular cartilage is a dense connective tissue without nerves, blood vessels, and lymph. It plays a load-bearing, buffering, and protecting role in joint movement (Carballo et al., 2017). Chondrocytes are the only cell type (accounting for 1%) in cartilage tissue and secrete growth factors and enzymes to regulate extracellular matrix (ECM) synthesis. They further embed themselves in ECM to form cartilage (Jiang and Tuan, 2014). The major ECM components, collagen II and aggrecan (ACAN), are classic markers of chondrocytic phenotype (Chijimatsu and Saito, 2019). The ECM network is responsible for absorbing mechanical stress of articular cartilage, promoting chondrocyte adhesion and regulating intracellular signal transduction.

**FIGURE 1:**
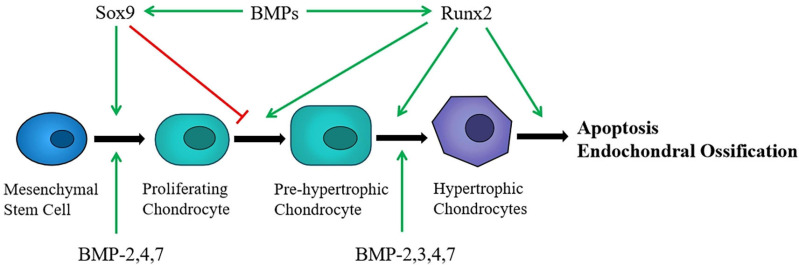
Chondrocytes originate from MSCs and undergo a series of differentiation processes. Sox9 induces MSCs differentiate into chndrocytes and promotes proliferation, whereas Runx2 stimulates chondrocyte hypertrophic. BMPs affect certain stages of chondrocyte differentiation and regulate the expression of Sox9 and Runx2.

Chondrocytes originate from bone marrow mesenchymal stem cells (BMSCs). First, aggregated BMSCs are capable of differentiating into chondroprogenitor cells. Then these chondroprogenitor cells become chondrocytes that undergo a series of differentiation processes and develop into hypertrophic chondrocytes (Figure 1). Finally, with the cartilage matrix partially calcified, chondrocytes are gradually replaced by osteoblasts after apoptosis and endochondral ossification is performed. Although chondrocyte hypertrophy and apoptosis are natural processes of endochondral ossification, it will accelerate the progress of osteoarthritis (OA) when cartilage is damaged (Rim et al., 2020). Besides, the growth and differentiation regulation system of chondrocytes cultured *in vitro* is extremely prone to imbalance, which can easily lead to cell aging and dedifferentiation. The process of chondrocyte dedifferentiation is accompanied by fibrous phenotype changes, decreased expression of collagen II, and increased expression of collagen I, matrix metalloproteinase 13 (MMP-13), and nitric oxide synthase (NOS) (Parreno et al., 2017; Charlier et al., 2019).

With the rapid development of transportation and sports and the acceleration of aging progress in human society, the incidence of joint cartilage trauma, strain, and degenerative diseases increased every year. Cartilage regeneration and repair capabilities are very limited. Once the cartilage is damaged, it is almost impossible to self-heal and may even degenerate. The treatment of cartilage injury is a difficulty in orthopedics, and the core of repairing cartilage is mainly by promoting chondrocyte proliferation and ECM synthesis (Richter et al., 2016). The process of chondrocytes in growth, metabolism, and differentiation is complicated. Several cytokines and cellular signals interact to regulate chondrocyte function and maintain cartilage homeostasis (Kozhemyakina et al., 2015; Liu et al., 2017; Fischer et al., 2018). It is of great significance to understand how the chondrocyte growth and development are affected by the interaction of key regulatory factors and cell signals.

## Key Regulatory Factors Regulating Chondrocyte Proliferation and Differentiation

### Sox9 Can Maintain Chondrocytes Phenotype and Inhibit Chondrocyte Hypertrophy

SRY-box 9 protein (Sox9) is an important transcription factor that mediates the differentiation of bone marrow mesenchymal stem cells (MSCs) into chondrocytes (Liu et al., 2017). It can be combined with collagen II and ACAN, then activate its own gene expression and induce chondrocyte proliferation and ECM synthesis (Akiyama et al., 2002; Soetjahjo et al., 2018). A recent study has shown that glutamine can control chondrogenic gene expression, protect chondrocyte survival, and promote chondrocyte proliferation and ECM synthesis. The realization of these effects depends on Sox9 stimulating glutamine metabolism (Stegen et al., 2020). In addition, several experimental studies have confirmed that Sox9 abundantly existed in cartilage progenitor cells and chondrogenic cells, which is a necessary condition for maintaining the chondrocytes phenotype (Zhao et al., 1997). Consecutively, Sox9 inhibits the differentiation of chondrocytes into pro-hypertrophic chondrocytes and does not participate in the further differentiation of hypertrophic chondrocytes at the end stage (Akiyama et al., 2004) (Figure 1), and then the expression of Sox9 is turned off. After that, another transcription factor Runx2 begins to be expressed (Yamashita et al., 2009).

### Runx2 Is Essential for Regulation of Chondrocyte Hypertrophy and Differentiation

Runt-related transcription factor 2 (Runx2) is essential for mediating chondrocyte maturation. The expression of Runx2 is low in proliferating chondrocytes, while it increased in pre-hypertrophic chondrocytes, and further increased in hypertrophic and terminal differentiated chondrocytes (Chen et al., 2014; Komori, 2017). Experimental study indicated that Runx2 regulates the expression of collagen X in hypertrophic chondrocytes, thus promoting endochondral ossification (Ding et al., 2012). There are also research that reported that maturation of chondrocytes was delayed in the Runx2 knockout mice (Yoshida et al., 2004; Takarada et al., 2013). These findings suggested that Runx2 can positively regulate chondrocyte maturation and endochondral ossification (Figure 1). Based on the effect on chondrocytes, Runx2 can be used as a target to regulate the differentiation and apoptosis of chondrocytes (Jiang et al., 2020). In addition, the expression of Runx2 in osteoarthritis chondrocytes is significantly higher than that of normal chondrocytes (Kamekura et al., 2006). With the decrease of Runx2, the progression of osteoarthritis is slowed down. As a result, Runx2 is a vital factor for chondrocyte maturation and participates in the pathogenesis of osteoarthritis.

### BMPs Is Involved in the Regulation of Chondrogenic Differentiation and Endochondral Ossification

Bone morphogenetic proteins (BMPs) are involved in almost all processes related to skeleton development (Wu et al., 2007), which belong to the TGF-β superfamily. As an osteo-chondrogenic factor, it positively regulates chondrocyte differentiation and endochondral ossification *via* transfer BMP signal from plasma membrane receptors to nucleus through Smad-dependent pathways and non-Smad-dependent pathways (Wan and Cao, 2005; Zhou et al., 2016; Chen et al., 2020). Smads, as a series of downstream effectors of Smad signaling pathways, is classified into three subgroups, a common-partner Smad (Co-Smad), receptor-regulated Smads (R-Smads), and inhibitory Smads (I-Smads). In Smad-dependent signaling pathways, the BMP-specific R-Smads (Smad1, 5, 8) were phosphorylated after BMPs binds to the receptors and subsequently form complexes with Co-Smad (Smad4). Then the complexes transferred to the nucleus to regulate the transcription of targeted genes (such as Sox9, Runx2) (Wu et al., 2007; Papathanasiou et al., 2012). Non-Smad-dependent signaling pathway, namely p38/mitogen-activated protein kinase (MAPK) signaling pathway, facilitates differentiation of mesenchymal cells into chondrocytes by activating Runx2 (Wu et al., 2016).

It is known that several BMPs play critical roles in maintaining cartilage homeostasis, such as BMP2, BMP3, BMP4, and BMP7 (Figure 1). In the interim, BMP2, BMP4, and BMP7 may induce the chondrogenic differentiation *via* regulating the expression of Sox9 and stimulate endochondral ossification through regulating the transcription of Runx2 (Liao et al., 2014; Wu et al., 2016; Zhou et al., 2016; Thielen et al., 2019). In addition, BMP3 can promote the maturation of terminal hypertrophic chondrocytes (Gamer et al., 2009). Futhermore, BMPs can promote the accumulation of mesenchymal cells and proliferation of chondrocyte by up-regulating the expression of the Wnt, Notch, and PI3K/AKT/mTOR signaling (Kobayashi et al., 2005; Zhang et al., 2019).

As the key transcription factors and growth factors for the growth and development of chondrocytes, Sox9, Runx2, and BMPs are regulated by multiple signal cascades. Next, we will focus on discussion of the relationship between signal pathways (Wnt, Ihh, TGF-β, FGF, Notch) and these factors.

## Cooperation Between Signal Pathways and Cytokines During Cartilage Development

### Wnt Signaling Pathway Interacts With Other Signaling Molecules to Regulate Chondrocyte Proliferation and Differentiation

**FIGURE 2:**
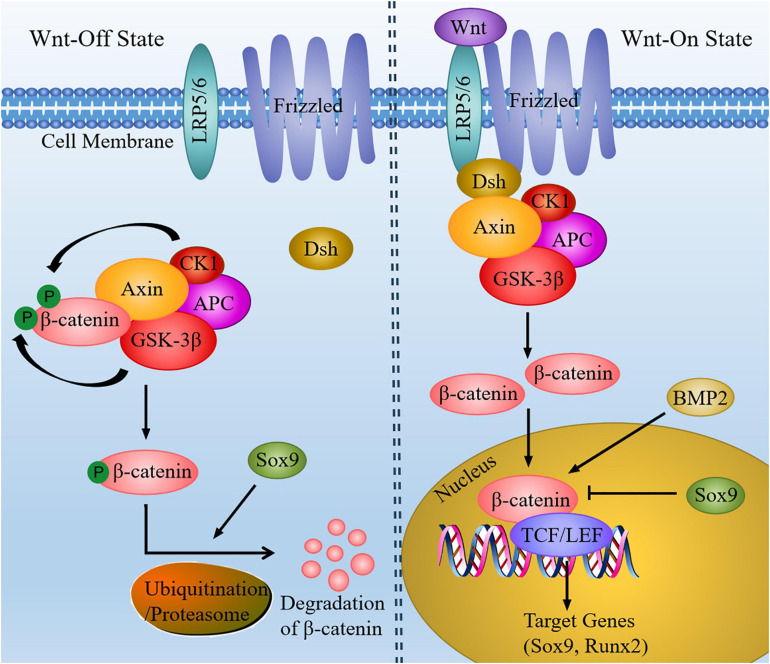
The canonical Wnt signaling pathway in chondrocytes. In the absence of Wnt, β-catenin could be phosphorylated by CK1 and GSK-3β. When Wnt signaling is activated, Wnt ligands combine with Frizzled and LRP5/6, and then recruit Dsh and destruction complex. So that the level of cytoplasmic β-catenin is increased. Subsequently, β-catenin enter the nucleus and bind to TCF/LEF to regulate the transcription of target genes (Sox9 and Runx2). In addition, BMP2 can increase the expression of nuclear β-catenin, whereas increased expression of Sox9 can inhibit β-catenin signaling and facilitate degradation of β-catenin.

Wnt family proteins are a sort of secreted glycoproteins that functions through autocrine or paracrine. Wnt signaling is transmitted by canonical Wnt signaling pathway (β-catenin-dependent pathway) and non-canonical Wnt signaling pathway (β-catenin-independent pathway), thereby regulating various biological processes (Clevers and Nusse, 2012). The non-canonical Wnt signaling pathway basically includes the planar cell polarity pathway (PCP) and Wnt/Ca^2+^ pathway, in addition to mitogen-activated protein kinase (MAPK), inositol triphosphate (IP3)-intercellular calcium, and c-Jun N-terminal kinase (JNK), which are activated independently of β-catenin, leading to cytoskeleton reorganization, chondrocyte stacking and different phenotypic responses. In canonical Wnt signaling pathway, Wnt proteins can combine with the seven transmembrane Frizzled protein receptor and the low-density lipoprotein receptor-related receptor 5/6 (LRP5/6), resulting in the recruitment of cytoplasmic protein Disheveled (Dsh) and destruction complex [the complex includes adenomatous polyposis coli (APC), glycogen synthesis kinase-3β (GSK-3β), Axin, and casein kinase 1 (CK1)]. After that, the phosphorylation of β-catenin by destruction complex is suppressed, thus causing β-catenin accumulation in the cytoplasm to become free β-catenin. Accordingly, free β-catenin move into the nucleus and form complexes with T cell factor/lymphoid enhancer factor (TCF/LEF), thereby controlling the transcription of target genes and activating the canonical Wnt/β-catenin signaling pathway (Figure 2). As the most important component of the canonical Wnt signaling pathway, β-catenin signal can control the differentiation of mesenchymal progenitor cells. If β-catenin is inactivated, such as β-catenin gene is knocked out in embryos, it will cause ectopic chondrogenesis (Day et al., 2005).

There are 19 members of the Wnt protein family. The canonical and non-canonical Wnt signaling regulates chondrocyte growth and metabolism *via* different Wnts. In this article, we focus on discussing the Wnt proteins which are only relevant to cartilage development. First, Wnt-3a can promote chondrocyte proliferation through Wnt/β-catenin signaling and induce chondrocyte differentiation *in vivo* through Wnt/Ca^2+^ signaling (Nalesso et al., 2011). Besides, Wnt-3a is considered to induce hypertrophy and differentiation of chondrocytes and participate in OA development (Bertrand et al., 2020), whereas Wnt-3a/β-catenin signaling could be inhibited by overexpression of Wnt-16, leading to reduced chondrocyte apoptosis (Yan et al., 2020). Second, Wnt-4 stimulates chondrocytes differentiation, and its overexpression will reduce the proliferation capacity of chondrocytes and accelerate the maturation (Zwaka et al., 2007). Next, Wnt-5a mediates non-canonical Wnt signaling to promote chondrocyte differentiation and inhibits the expression of collagen II (Kawakami et al., 1999), while Wnt-11 plays the opposite role (Ryu and Chun, 2006). However, Wnt-1 and Wnt-7a block chondrocyte differentiation through inhibiting the aggregation of mesenchymal stem cells (Rudnicki and Brown, 1997). Moreover, the decrease of Wnt-7a expression and the increase of Wnt-5a expression can promote the dedifferentiation of chondrocytes (Sassi et al., 2014b). Last, Wnt-9a promotes chondrocyte maturation and regulates Indian hedgehog (Ihh) protein expression (Spater et al., 2006). It has been shown that the lack of wnt-5b and wnt-9a resulted in delayed endochondral ossification (Ling et al., 2017).

Wnt signaling can also work with other signaling molecules to mediate chondrocyte development. For instance, Wnt signaling pathway mediated by β-catenin may be inhibited along with increased expression of sox9 (Topol et al., 2009). Several experiments have found that mutant mice of Sox9-overexpression in chondrocytes exhibited chondrodysplasia, which is similar to β-catenin-null mutant mice. Among the two mutant mice mentioned above, the differentiation of hypertrophic chondrocytes and endochondral ossification is delayed (Akiyama et al., 2004). As a result, the activation of β-catenin-dependent promoters is inhibited by Sox9 and the degradation of β-catenin is facilitated through the ubiquitination/proteasome pathway.

According to the study, Wnt/β-catenin signaling is able to mediate chondrocyte hypertrophy through inducing type X collagen alpha 1 (Col10a1) upregulation and activating Runx2 expression (Dong et al., 2006). Besides, BMPs also play critical roles in chondrocyte hypertrophy. Previous study has shown that Wnt/β-catenin signaling could be mediated by BMP2 to regulate chondrocyte hypertrophy. The molecular mechanism has revealed that BMP2 increased the protein level of nuclear β-catenin in chondrocytes (Papathanasiou et al., 2012). It has been reported that Wnt act upstream of Ihh in growth plate chondrocytes. Moreover, Wnt signaling can drive the up-regulation of Ihh and BMP signals during the process of endochondral MSC differentiation, thus promoting chondrocyte hypertrophy, cartilage mineralization and bone metastasis (Diederichs et al., 2019). Furthermore, ERK1/2 signal can be activated by non-canonical Wnt signal, thus inducing the loss of chondrocytes phenotype and promoting chondrocytes dedifferentiation (Xie et al., 2018). To sum up, the interaction between Wnt signaling pathway and other signaling molecules can mediate chondrocyte proliferation, differentiation and maturation. Although the function of Wnt signaling pathway has not been fully explained, there is still a lot of room for discovering more possible mechanisms.

### Ihh Signaling Pathway Promotes Chondrocyte Proliferation and Inhibits Its Differentiation Through Ihh-PTHrp Feedback Loop

**FIGURE 3:**
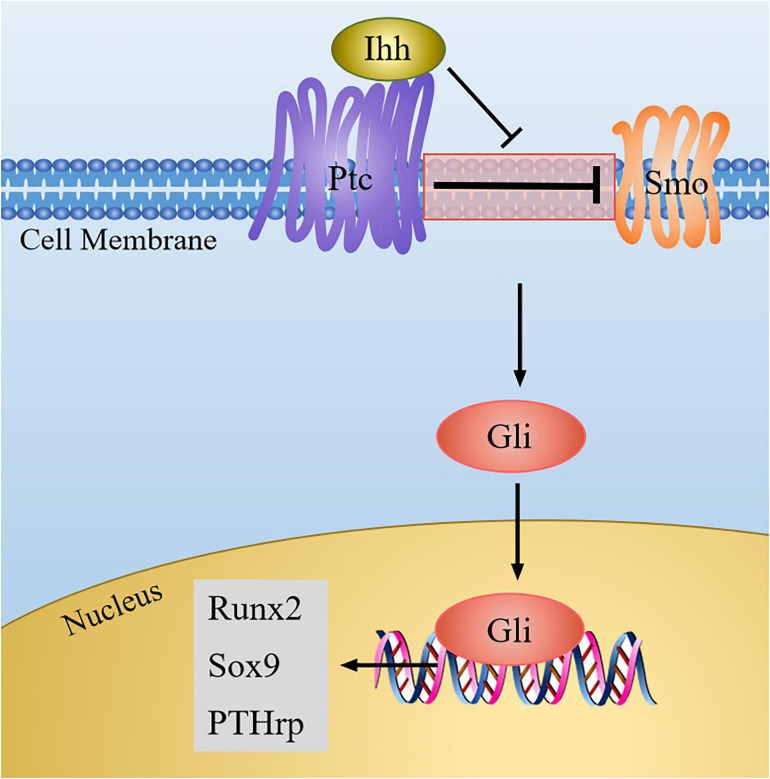
The Ihh signaling pathway in chondrocytes. When Ihh signaling is produced, it binds to Ptc, and then the inhibition of Ptc on Smo is relieved, which result in the activation of Gli. Next Gli enters the nucleus to regulate the expression of target genes (Sox9, Runx2, PTHrp).

Ihh belongs to the vertebrate hedgehog protein family. Once the Ihh signal is produced, its amino-terminal part can combine with the transmembrane protein Patched (Ptc), and then the suppression of Ptc on smoothened (Smo, a class of G protein-coupled multichannel membrane protein) is blocked. Thus, Smo was activated, and the signal is transmitted to downstream effectors, causing the activation of Gli family genes, which are all transcription factors mediating the hedgehog pathway and originally isolated from human glioblastoma cells (Yang et al., 2015). Soon thereafter, Gli enters into the nucleus and initiates the expression of downstream genes, such as parathyroid hormone-related protein (PTHrp) (Figure 3).

**FIGURE 4:**
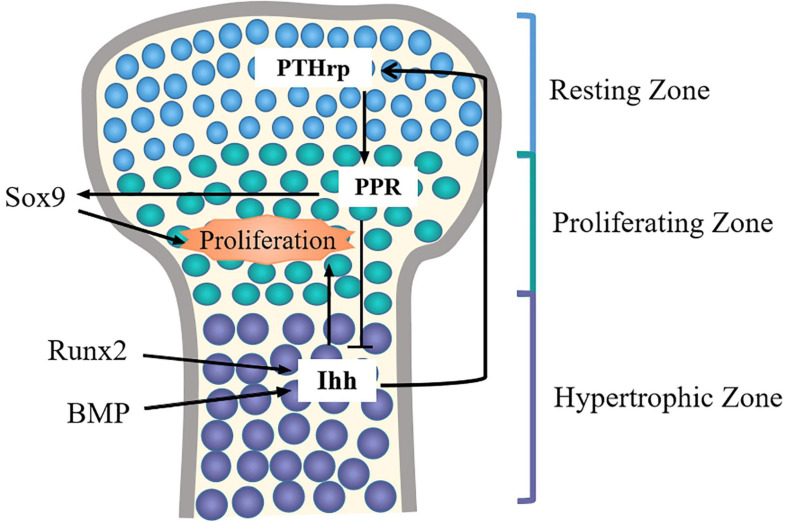
The Ihh-PTHrp feedback loop in chondrocytes. In articular cartilage, Ihh is expressed in pre-hyertrophic chondrocytes and hyertrophic chondrocytes, it also can promote chondrocytes proliferation. Ihh stimulates the expression of PTHrP in periarticular and perichondral chondrocytes to promote chondrocytes proliferation *via* increasing Sox9 activity. PTHrP in turn inhibits the hypertrophy of chondrocytes *via* PPR, and Ihh expression is turned off. BMP and Runx2 can induce the expression of Ihh.

Ihh has been indicated that it may control chondrocyte proliferation and differentiation through Ihh-PTHrp negative feedback loop (Vortkamp et al., 1996; Kindblom et al., 2002) (Figure 4). On the one hand, PTHrp is induced by Ihh signal and diffused into growth plate region to promote chondrocyte proliferation. Meanwhile, PTHrp in turn acts on PTH/PTHrp receptors (PPR) to block chondrocyte terminal differentiation, thus maintaining the proliferative of chondrocytes and turning off the expression of Ihh (Zhao et al., 2002; Sasai et al., 2019). On the other hand, when PTHrP is not enough to stimulate chondrocyte proliferation, chondrocytes begin to secrete Ihh, and the expression level of PTHrP is upregulated, thereby promoting the proliferation of chondrocytes and inhibiting the terminal differentiation of hypertrophic chondrocytes. When Ihh signal is absent, the expression of PTHrp is reduced, which accelerates chondrocyte hypertrophy (Yang et al., 2015).

Additionally, Ihh signaling and other signaling molecules jointly regulate chondrocyte development. For example, the activation of PPR may raise the phosphorylation of protein kinase A (PKA), thus phosphorylating Sox9 so that the differentiation process of chondrocytes is delayed (Kronenberg, 2006; Nowak-Solinska et al., 2013). In addition, Runx2 can directly bind to the promoter of the Ihh and induce up-regulation of Ihh levels, and consequently, the maturation and differentiation of chondrocytes is promoted (Yoshida et al., 2004). Intriguingly, a recent study found that desert hedgehog (Dhh), another member of the hedgehog family, can promote the expression of Col X and Runx2 to promote chondrocyte hypertrophy (Ma et al., 2020). This suggests that Ihh and Dhh signals may synergistically promote the differentiation of chondrocytes. Furthermore, BMP can induce the Ihh-PTHrp signaling by increasing the expression of Ihh to mediate chondrocyte differentiation (Retting et al., 2009). When there was no hedgehog signal inputs, BMP might enhance the formation of ectopic chondrocytes in the perichondrium (Hojo et al., 2013). In addition, a study showed that total flavonoids of Rhizoma can simultaneously up-regulate the expression of BMP/Runx2 and Ihh/PTHrp to repair the damage caused by thiram on chondrocytes and improve cell viability (Yao et al., 2020). Therefore, Ihh signaling and other signaling molecules together determine the proliferation and differentiation of the chondrocytes. It is warranted to further explore the regulatory effects of other signaling molecules on Ihh signaling pathway.

### TGF-β Signaling Pathway Regulates the Expression of Transcription Factors and Growth Factors to Control Chondrocyte Proliferation and Differentiation

**FIGURE 5:**
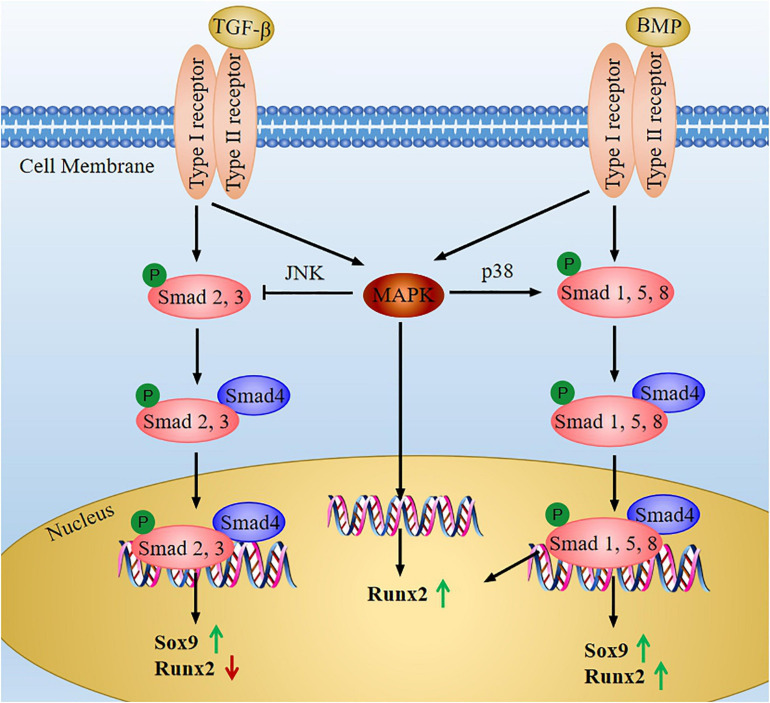
The TGF-β and BMP signaling pathway in chondrocytes. When TGF-β or BMPs bind to type II receptor, type I receptor is transphosphorylated, which activate Smad-dependent signaling or non-Smad-dependent signaling (MAPK signaling). In the Smad-dependent signaling, R-Smads (the TGF-β-specific R-Smads is Smad2, 3 and the BMP-specific R-Smads is Smad1, 5, 8) are phosphorylated, and then it forms complexes with Co-Smad (Smad4). Next the complexes transfer into nucleus to regulate the expression of Sox9 and Runx2. MAPK signaling can phosphorylate Runx2 to increase its transcription activity, and it also can activate JNK and p38 kinases to change the balance between Smad2/3 and Smad1/5/8.

Transforming growth factor (TGF-β) is a kind of polypeptide growth factor. It involves many biological processes, such as embryonic development, inflammation, cell growth, immune response, and carcinogenesis (Morikawa et al., 2016). The TGF-β superfamily includes TGF-β, BMPs, activin, growth and differentiation factors (GDFs), and nodal. After TGF-β superfamily proteins combine with type II receptor, type I receptor (also called active receptor-like kinases, ALKs) is activated by type II receptor, thus resulting in phosphorylation of R-Smads or activation of MAPK cascade (Grafe et al., 2018) (Figure 5). On the one hand, in Smad-dependent TGF-β signaling pathway, the complexes composed of phosphorylated R-Smads (Smad2, 3) and Co-Smad (Smad4) transfer into nucleus, thereby regulating downstream target gene expression, such as Sox9 and Runx2 (van der Kraan et al., 2009). So that the proliferation and differentiation of chondrocytes could be controlled by different transcription factors (Wu et al., 2016). On the other hand, non-Smad-dependent pathway (MAPK pathway) induced by TGF-β can mediate cartilage homeostasis (Thielen et al., 2019). The phosphorylation of MAPK may activate JNK and p38 kinases, which changes the balance between TGF-β signaling and BMP signaling, so it will accelerate the terminal differentiation of chondrocytes (Kraan et al., 2010).

Herein, we will illustrate the current understanding that the interaction between TGF-β and key regulatory factors (for example, Runx2, Sox9, BMP) affects cartilage development. The expression of Runx2 is suppressed by the activation of the TGF-β/Smads signaling, thereby reducing chondrocyte ECM degradation (Xiao et al., 2018; Janssen et al., 2019). On the contrary, Sox9 protein level can be stabilized by TGF-β, and both of them synergistically protect chondrocytes’ function (Chavez et al., 2017). In addition, there is recent research that studied the mechanism of salidroside to promote chondrocyte proliferation, and it found that the expression of Sox9 was up-regulated by salidroside, and the expression of TGF-β and Smad3 was up-regulated (Sun et al., 2020). As a member of the same protein family, TGF-β1 may induce the expression of BMP2 to promote the proliferation of chondrocytes. Contrarily, BMP2 may inhibit the activation of TGF-β-induced Smad signal to suppress the terminal differentiation of chondrocytes. Just like Ihh-PTHrp, TGF-β and BMP form a feedback loop to regulate chondrocyte development (Wu et al., 2016).

TGF-β signaling can also participate in the signal transduction of other signaling pathways. Current evidence indicated that activation of TGF-β signal is accompanied by increased expression of Wnt protein and its receptor, as well as the aggregation of β-catenin in the nuclear (Zhou et al., 2004), the interaction of TGF-β and Wnt signaling pathway stimulated chondrocyte differentiation. Furthermore, TGF-β can also promote the proliferation and differentiation of chondrocytes *via* the Notch-Sonic hedgehog (Shh)-Foxa pathway (Ma et al., 2019). Accordingly, these investigations revealed that TGF-β signaling pathway interacts with other cytokines to control chondrocyte proliferation and differentiation.

### FGF Signaling Pathway Mediates Chondrocyte Development *via* Regulating Cytokines Expression

**FIGURE 6:**
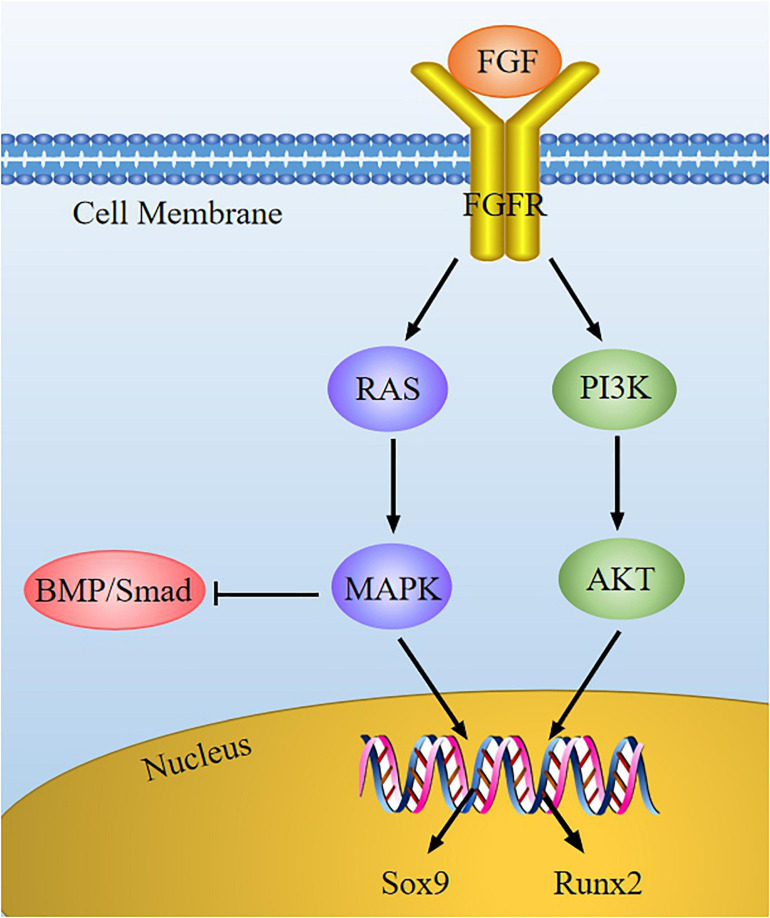
The FGF signaling pathway in chondrocytes. FGF combines with FGFR to activate the downstream cascade (RAS-MAPK and PI3K-AKT), and then the expression of Sox9 and Runx2 is regulated. FGF can suppress BMP/Smad signaling *via* MAPK signaling in chondrocytes development.

Fibroblast growth factor (FGF) is a sort of peptide that can promote cell mitosis and angiogenesis. In the skeletal system, FGF signaling is related to homeostasis regulation of the cartilage environment and the occurrence of cartilage degenerative diseases. FGF may bind to specific receptor (FGFR), and then the tyrosine kinase domain of FGFR located inside the cell membrane is activated, thus activating downstream signals (such as RAS-MAPK and PI3K-AKT) to mediate chondrocyte development (Figure 6).

Several FGFs and FGFR are considered to be the important regulator of chondrocyte development. Multiple studies have shown that FGF2, FGF9, and FGF18 can facilitate mesenchymal stem cells differentiation into chondrocytes, stimulating chondrocyte proliferation and ECM synthesis (Chuang et al., 2010; Ellman et al., 2013; Cinque et al., 2015; Correa et al., 2015; Shu et al., 2016). Moreover, FGF23 is highly expressed in osteoarthritis chondrocytes, and its up-regulation may induce chondrocyte hypertrophy (Orfanidou et al., 2009). Many studies have indicated that cartilage hypoplasia is manifested in FGFR1/2 knockout mice (Karuppaiah et al., 2016), while the dwarfism is also induced in FGFR3-mutant mice on account of the activation of FGFR3 (Sahni et al., 2017). In addition, FGFR1/2 exists in proliferating chondrocytes, while FGFR3 exists in hypertrophic chondrocytes (Zhou et al., 2015). Therefore, it is generally believed that FGFR1/2 can promote the proliferation and differentiation of chondrocytes, whereas FGFR3 cannot, and FGFR3 can also facilitate the apoptosis of chondrocytes.

Based on literature reports, we proposed our understanding of how FGF signaling interacts with other cytokines to intervene chondrocyte development. On one hand, FGFs can enhance Sox9 expression to promote chondrocyte proliferation *via* the MAPK pathway (Murakami et al., 2000). Activated FGFR3 raises the transcriptional activity of Sox9, causing the suppression of chondrocyte hypertrophy and reduction of chondrogenesis (Zhou et al., 2015). On the other hand, FGF2 may improve the activity of Runx2 *via* MEK/ERK pathway, leading to chondrocyte differentiation (Wang et al., 2004). As previously described, BMP signaling can promote chondrocyte proliferation, whereas FGF2 induces chondrocyte hypertrophy and differentiation. Furthermore, the FGF signaling is stimulated when the BMP signaling is inhibited by Noggin (BMP antagonist) (Minina et al., 2002; Otsuki et al., 2010). Therefore, FGF and BMP play an opposite role in regulating chondrocyte proliferation, hypertrophy, and differentiation. These suggest that we can regulate the expression of key regulatory factors through FGF signaling to maintain cartilage homeostasis.

### Proper Notch Signaling Is Vital for Chondrogenesis and Normal Chondrocyte Differentiation *via* Modulating Key Regulatory Factors Expression

**FIGURE 7:**
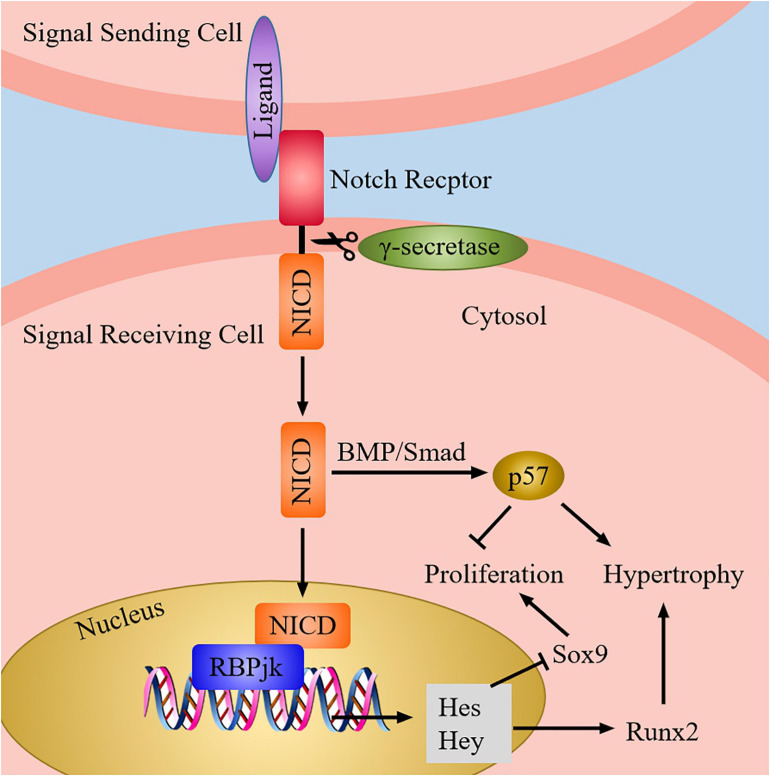
The Notch signaling pathway in chondrocytes. When Notch signaling is produced, Notch ligand of adjacent cells bind to Notch receptor, then the receptor is cleaved by γ-secretase to release the NICD. Next, NICD enters the nucleus and interact with RBPjk to induce the target genes expression (Hes and Hey). Afterward, the expression of Sox9 is inhibited, whereas the expression of Runx2 is increased. Besides, Notch also can induce the expression of p57 *via* BMP/Smad signaling to promote chondrocyte hypertrophy and suppress chondrocyte proliferation.

Notch signaling pathway consists of ligands, Notch receptors, and CSL (CBF-1, Suppressor of hairless, LAG) proteins, and it can regulate chondrocyte proliferation and differentiation *via* the interaction of the adjacent cells (Zieba et al., 2020). After the ligand binds to the receptor, the Notch signaling is activated, which causes the cleavage of extracellular domain of receptor. Next, the receptor is cleaved three times to release Notch intracellular domain (NICD). When the canonical Notch signaling is activated, NICD is transferred into the nucleus to interact with recombination signal binding protein-jk (RBPjk) which is on DNA, and then form a transcription complex. As a result, the transcription factor induces the expression of downstream target genes, which includes the Hairy enhancer of split (Hes) and Hes-related with YRPW motif (Hey) family genes, thereby affecting chondrocyte proliferation and differentiation (Hosaka et al., 2013) (Figure 7).

Experiments have proved that overexpression of Notch can inhibit the expression of Sox9, collagen II, and ACAN in chondrocytes, thus decreasing chondrocyte proliferation and suppressing hypertrophic chondrocyte differentiation (Chen et al., 2013). On the contrary, absence of Notch signaling may increase Sox9 expression, causing chondrocyte proliferation and hypertrophy (Mead and Yutzey, 2009). Moreover, activation of Notch1 can upregulate the expression of Sox9 in embryonic MSCs, which induce chondrogenic (Haller et al., 2012). Therefore, these dates supported the conclusion that appropriate level of Notch signaling is crucial for chondroprogenitor cell proliferation and normal hypertrophic chondrocyte differentiation through modulating Sox9 expression. In addition, MMP-13 is the most effective collagen II degrading enzyme in the family of matrix metalloproteinases (MMPs); it can be regulated and controlled by Notch signaling *via* the activation of Runx2 (Xiao et al., 2019), thereby promoting the degradation of collagen II in chondrocytes and facilitating the differentiation of hypertrophic chondrocytes (Blaise et al., 2009; Sassi et al., 2014a). Notch can also induce cell cycle arrest and promote chondrocyte hypertrophy through upregulation of p57 expression which is mediated by BMP/Smad signaling (Shang et al., 2016). Hence, these findings indicates that Notch signaling pathway is necessary for the chondrogenic differentiation and normal chondrocyte development *via* regulating transcription factors and growth factors expression.

## Conclusion

Articular cartilage is in the physiological environment with various biochemical and biophysical stimulation signals. The process of chondrocyte proliferation and differentiation is affected by key factors (such as Sox9, Runx2, BMPs) and cell signals (such as Wnt, TGF-β, FGF, Ihh, and Nocth), thereby promoting the synthesis of ECM and expressing the characteristics and functions of chondrocytes. The regulation of chondrocyte growth and maturation does not depend on the single role of a signal molecule, but the interaction and coordination of several molecules. Although a lot of scientific work has been done on the cartilage development both in experimental and in theoretical fields, the exploration is ongoing. We reviewed the various signaling factors that regulate cartilage development and revealed the relationship among them, hoping to provide more ideas for the treatment of cartilage damages.

## Author Contributions

HC and X-NT wrote the main part of the manuscript and took part in the drawing. SH, R-QL and L-HP was responsible for the collection, sorting, and analysis of documents. PW and Y-ML selected the topic and also wrote the parts of the manuscript. All authors have read and agreed to the published version of the manuscript.

## Conflict of Interest

The authors declare that the research was conducted in the absence of any commercial or financial relationships that could be construed as a potential conflict of interest.
